# Identification of the causative agent of cutaneous leishmaniasis in Chichaoua province, Morocco

**DOI:** 10.1051/parasite/2012191081

**Published:** 2012-02-15

**Authors:** M. Rhajaoui, F. Sebti, H. Fellah, M.Z. Alam, A. Nasereddin, I. Abbasi, G. Schönian

**Affiliations:** 1 Département de parasitologie, Institut National d’Hygiène Rabat Morocco; 2 Institut fuer Mikrobiologie und Hygiene, Charité Universitaetsmedizin Berlin Germany; 3 Department of Biology, University Al-Quds Jerusalem Palestine

**Keywords:** leishmaniasis, *Leishmania tropica*, ITS1-PCR, Morocco, leishmaniose, *Leishmania tropica*, ITS1-PCR, Maroc

## Abstract

Cutaneous leishmaniasis (CL) in Morocco is caused by three species, *Leishmania major, L. tropica* and *L. infantum*. CL has been known in Chichaoua province since 2000. Using DNA extracted from microscopic slides and parasite cultures, collected in the years 2006 and 2009, we identified for the first time *L. tropica* as the causative agent of CL in this region. Species identification was achieved by performing the ITS1-PCR-RFLP approach. By using this method it was possible to identify parasites in Giemsa stained slides containing less than five parasites per oil-immersion field even they were conserved for up to four months.

## Abstract (fr)

La leishmaniose cutanée (LC) dans la province de Chichaoua est connue depuis l’an 2000. Cette étude montre pour la première fois l’espèce parasitaire circulante dans cette province. L’identité du parasite a été déterminée sur des lames colorées et des cultures de parasites en utilisant l’ITS1-PCR, suivie de la digestion par l’enzyme HaeIII. Le résultat a montré que *Leishmania tropica* est l’agent responsable de la LC. Par ailleurs, une corrélation entre le nombre de parasites par lame et la positivité du test moléculaire a été réalisée et a montré que l’ITS1-PCR permet d’identifier des parasites dans les lames colorées au Giemsa contenant moins de cinq parasites par champs et même si elles étaient conservées jusqu’à quatre mois.

In Morocco, three species of *Leishmania* causing human cutaneous leishmaniasis (CL) are endemic (Rhajaoui, 2010). *Leishmania major*, responsible for zoonotic CL was localized in areas south of the Atlas Mountains (Rioux *et al.*, 1986) where regular epidemics with more than 2,000 cases were reported (Ministère de la Santé, 2007). In the north of the country, some cases of CL due to *L. infantum* were observed (Rhajaoui *et al.*, 2007). CL caused by *L. tropica* has been reported and well documented (Marty *et al.*, 1989; Guessouss-Idrissi *et al.*, 1997). In Chichaoua province, first cases of CL were recorded in 2000 and it was considered as emerging epidemic focus (Ministère de Santé, 2001). It has been suggested that the disease there was caused by *L. tropica* and that transmission was by *Phlebotomus sergenti* (Guernaoui *et al.*, 2005). In this study, we have applied the ITS1 PCR-RFLP assay (Schönian *et al.*, 2003) to samples collected in the years 2006 and 2009 and showed for the first time that the causative agent of CL in Chichaoua province is indeed *L. tropica*. The capacity of the ITS1-PCR to identify parasite in different slides with different parasite burden was also evaluated.

## Materials and Methods

### Case detection

Chichaoua province located at 460 km southern of Rabat covers an area of 7,120 km2 with altitude ranging from 322 to 1,446 m above sea level. The vegetation is rare and mainly dominated by jujube plant, olive, carob and almond trees (Guernaoui *et al.*, 2005).

In 2006, 14 positive slides were obtained by dermal scraping of the active indurate margins of the lesions of 14 patients and were smeared on a slide for staining with Giemsa and examined microscopically for presence of amastigotes. Subsequently, *Leishmania* parasites were successfully cultured in NNN medium for the same 14 patients.

Three years later, in 2009, 34 slides from new cases were confirmed by microscopy as CL. After four months of isolation, the 34 slides were divided into four groups according to the average number of amastigotes counted in 10 oil-immesion fields (OIF) scanned randomly by two different blinded persons using the same light microscopy. The quantitative grading of parasitic density in slides is shown in Table I.

**Table I.** Quantitative grading of *Leishmania* amastigotes using stained slides and light microscopy.

Group	Number of slides	Average number of amastigotes per OIF
G1	10	> 100
G2	8	50 – 100
G3	8	10 – < 50
G4	5	5 – 10
	3	< 5

### Molecular identification

This analysis was performed for 48 *Leishmania* positive slides collected in 2006 and 2009 and 14 cultures maintained in NNN medium. Briefly, DNA was extracted from cultured parasites or positive slides using a phenol chloroform DNA extraction protocol (Meredith *et al.*, 1993, El Tai *et al.*, 2000), the internal transcribed spacer (ITS1) of the ribosomal RNA gene region was amplified and the PCR product was subjected to digestion with endonuclease *Hae*III. The entire RFLP product was loaded on agarose gels and analysed as previously described (Schönian *et al.*, 2003; Al-Jawabreh *et al.*, 2004). Reference strains of *L. tropica* (MHOM/AZ/1974/SAF-K27), *L. major* (MHOM/ IL/1967/Jericho) and *L. infantum* (MHOM/TN/1980/ IPT1) were used for comparison.

## Results

**Fig. 1.**
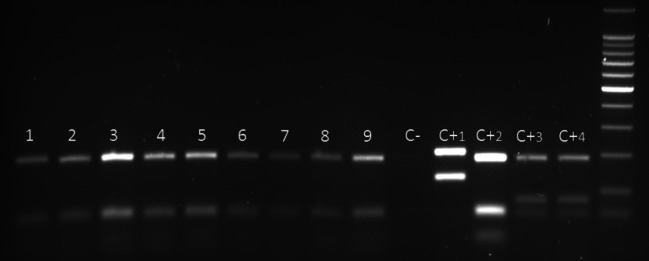
Application of RFLP analysis of the ITS1-PCR analysis on positive amastigotes slides from Chichaoua province in 2009. 1-7, samples from slides; C-, negative control; C+1, positive control of *L. major* (MHOM/IL/1967/Jericho); C+2, positive control of *L. tropica* (MHOM/AZ/1974/SAF-K27); C+3 and C+4, positive control of *L. infantum* (MHOM/TN/1980/IPT1).

The *Leishmania* parasites in all clinical samples and cultures analysed in this study were identified as *L. tropica* by their typical restriction profiles (Fig. 1). ITS1 PCR produced one single band, which was 300 bp in size. The RFLP analysis revealed two fragments (185 and 57 bp), which were specific for *L. tropica* as previously described (Schönian *et al.*, 2003). Furthermore, it could be shown that the ITS1- PCR-RFLP assay can detect and identify parasites in Giemsa stained slides containing less than five parasites per oil-immersion field.

## Discussion

This study provides the first direct evidence that CL in Chichaoua region is caused by *L. tropica*. Previous studies on leishmaniasis in this area have described the region as a focus of CL due to *L. tropica* solely based on epidemiological and clinical aspects (Guernaoui *et al.*, 2005). In fact, the clinical symptoms of CL were similar to those seen in foci of *L. tropica* in Taza and Zouagha My Yacoub, and differed clearly, both in form and size, from lesions observed in zoonotic leishmaniasis due to *L. major* identified in CL foci in southern Morocco. However, geographical origin of the parasites is an inadequate criterion in non-endemic areas as well as endemic regions where multiple species of *Leishmania* may coexist. Similarly, identification of the infecting species based on clinical symptoms can be problematic, since several species cause both visceral and cutaneous disease (Rhajaoui, 2010; Schönian *et al.*, 2003). Thus, as symptoms can vary and may be confused with other etiologic agents, diagnostic confirmation of *Leishmania* parasite is mandatory. Moreover, it was useful to identify the parasite circulating in Chichaoua region in 2006 and 2009 in order to see if *L. tropica* is indeed and the only causative agent for CL in this region.

As the risk patterns for CL have changed recently in several countries (Mihoubi *et al.*, 2008), the observation of *L. tropica* species from cutaneous lesions of patients living in the Chichaoua area suggests a modification of the epidemiology of CL in Morocco.

The emergence of CL due to *L. tropica* as an increasingly important public health problem in Morocco appears to be related to several factors. The most important of them are probably ecological and dermatographic changes (Ashford, 1999). The spread of the disease might also be facilitated by increasing urbanization processes in the settlements, that are usually overcrowded and provide inadequate housing and poor sanitations. Human and dog population movement could act as an important risk factor in spread of the disease. In fact, the high risk for *L. tropica* introduction in southern Italy was related to the importation of infected dogs from northern Africa and the presence of the vector (Gramiccia & Gradoni, 2005). This might also have lead to the emergence of CL in Chichaoua.

In this study, we showed also that *Leishmania* DNA could be efficiently extracted and amplified four months old Giemsa stained microscopic slides even these were not protected by a cover slip and contain less than five parasites in each OIF. ITS1-PCR based techniques represent a very sensitive method for the diagnostic of CL from these slides.

Indeed, our result should be considered for the national strategy to control the disease and to evaluate any future potential foci. The identification of *L. tropica* in cutaneous samples from patients in Chichaoua district is certainly only a first step. Previously, isoenzyme typing (Pratlong *et al.*, 1991; Dereure *et al.*, 1991) as well as multilocus microsatellite typing (Schwenkenbecher *et al.*, 2006) have shown high genetic diversity of Moroccan strains of *L. tropica* with different zymodemes/genotypes correlating with anthroponotic and zoonotic transmission cycles of the parasites present in the same CL foci. It should be analyzed next whether the disease in Chichaoua is due to a single or a variety of *L. tropica* genotypes because that would have consequences for the design of appropriate control measures. Deciphering the transmission factors in an era of climate change will require multicentre and multidisciplinary studies, focusing on reservoir, vectors, therapeutic efficacy, parasite species and strain identification. Local stakeholders and health authorities should be involved in such a network for disease surveillance.
